# Metastatic renal cell carcinoma to the pancreas, thyroid, & subcutaneous tissue 13 years after Radical nephrectomy: A case report

**DOI:** 10.1016/j.ijscr.2019.05.031

**Published:** 2019-05-28

**Authors:** Alaa A. Al Abdrabalnabi, Abdullah S. AlQattan, Shahad Algarni, Miral Mashhour, Mohammed Al-Qahtani

**Affiliations:** a King Fahd University Hospital, Imam Abdulrahman Bin Faisal University, Saudi Arabia; b Department of Histopathology, King Fahad Specialist Hospital-Dammam, Saudi Arabia; c Department of General Surgery, Hepatobiliary & Multi Organ Transplant, King Fahad Specialist Hospital-Dammam, Saudi Arabia

**Keywords:** Renal cell carcinoma, Renal cell carcinoma metastasis, Case report, Thyroid metastasis, Pancreas metastasis, Subcutaneous metastasis

## Abstract

**Introduction:**

Renal cell carcinoma (RCC) accounts for 3% of all adult malignancies and is known for its poor prognosis. At the time of diagnosis up to 30% of patients will present with metastasis. We report multiple metastases of RCC to rare sites; twice to pancreases, thyroid, twice to subcutaneous tissue, and the other kidney- occurring years after the radical nephrectomy.

**Case presentation:**

Our case is of a 51-year-old female who underwent a right radical nephrectomy for RCC. Upon 6 years post-operative surveillance for recurrence, multiple metastatic lesions were found in the head of the pancreas which were resected by a Whipple procedure. Four years later, she presented with a thyroid nodule and fine needle aspiration showed metastatic RCC. The patient underwent a total thyroidectomy. Months later, the patient presented with a right forearm mass. Biopsy showed metastatic RCC. Excision with negative margins was performed. A year later, a metastatic lesion was found in the tail of pancreas in addition to a recurrent metastatic mass over the right forearm. The patient underwent completion pancreatectomy and resection of the forearm mass. One year later, another metastatic lesion was found in the left kidney, for which the patient underwent left partial nephrectomy with negative margins.

**Conclusion:**

RCC is one of the most aggressive malignancies. A high index of suspicion is crucial to detect its metastasis. As some lesions maybe missed because they lack the classical radiological findings or are found in atypical locations, in addition to the latent recurrence of RCC metastasis, a lifelong follow-up is recommended.

## Highlights

• Renal cell carcinoma (RCC) is an aggressive cancer accounting for 3% of all malignancies.
• RCC has been reported to be one of the most common malignancies to cause solitary pancreatic metastasis.
• Metastasis to thyroid is reported to occur in 1% of patients, and can present as late as 20 years after the resection of the primary tumor.
• A high index of suspicion is crucial to detect RCC metastasis, in addition to life-long follow-up.

## 1 Introduction

This work has been reported in line with the SCARE criteria [1]. Renal cell carcinoma (RCC) is known for being an aggressive and often lethal cancer, accounting for around 3% of all malignancies. Metastasis from RCC develop in one-third of the patients, and in such cases the prognosis is extremely poor and the disease is regarded as fatal [2]. RCC is also characterized by long periods of recurrence and a tendency for unusual metastatic spread [[2], [3], [4]]. The metastatic pathway of RCC is not predictable partially owing to its complex lymphatic drainage, with the commonest sites of metastasis being the adrenals, lungs, brain, and liver [2].

## 2 Case presentation

**Fig. 1:**
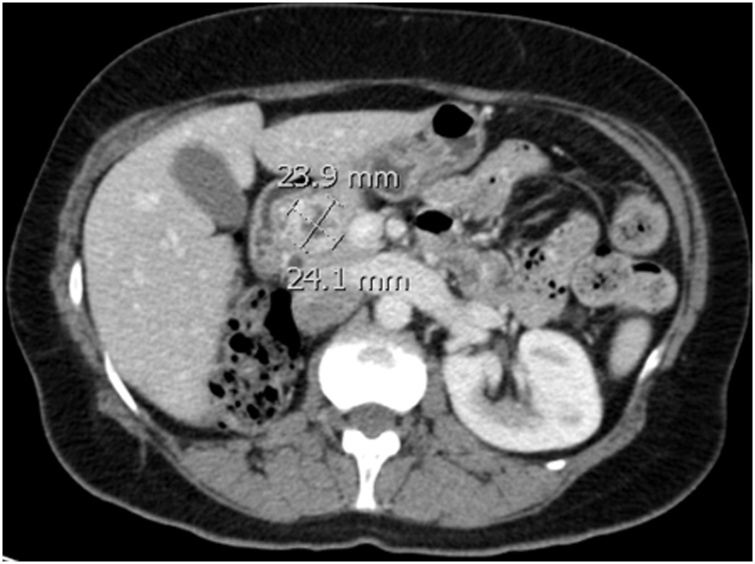
Axial view CT scan of the abdomen showing a metastatic lesion in the head of the pancreas.

**Fig. 2:**
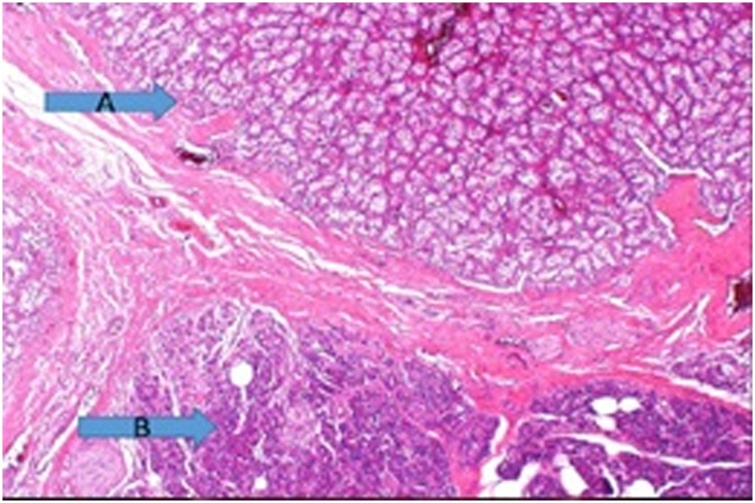
Histopathology of the head of pancreas showing RCC metastasis (**A**), in contrast to normal pancreatic tissue (**B**).

**Fig. 3:**
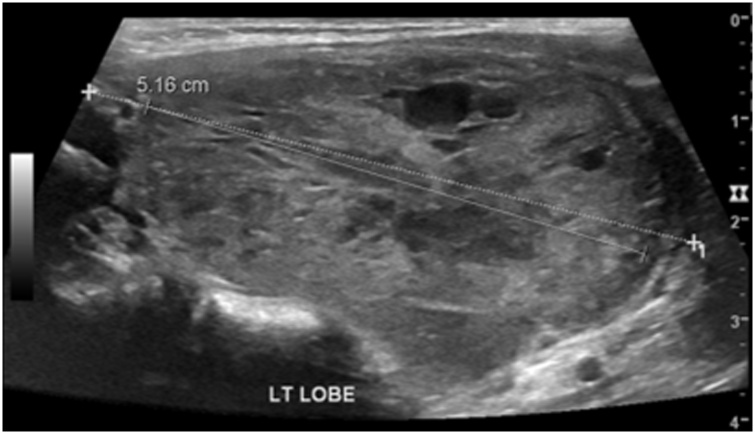
Thyroid ultrasound showing a nodule in the left lobe of the thyroid gland.

**Fig. 4:**
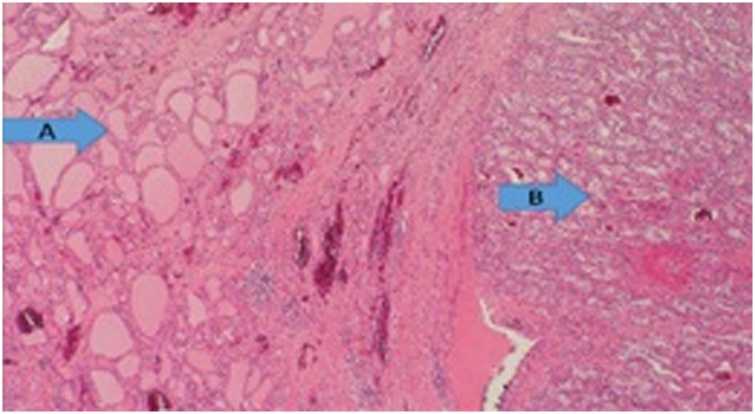
Histopathology of the thyroid gland showing normal glandular tissue (**A**), and a metastatic lesion consistent with RCC (**B**).

**Fig. 5:**
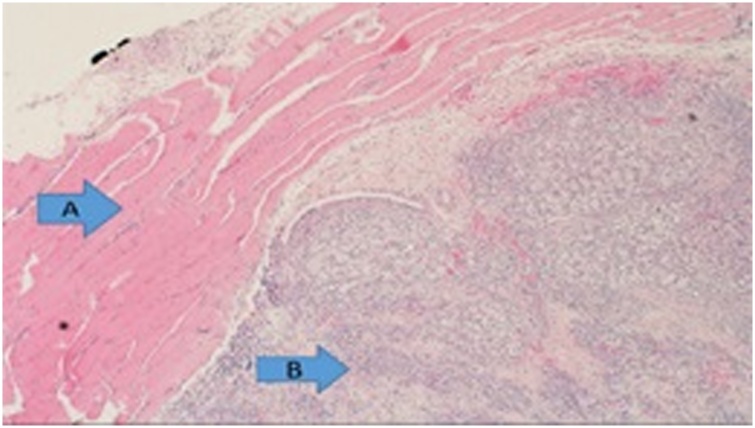
Histopathology showing subcutaneous tissue (**A**) with a lesion consistent with RCC metastasis (**B**).

**Fig. 6:**
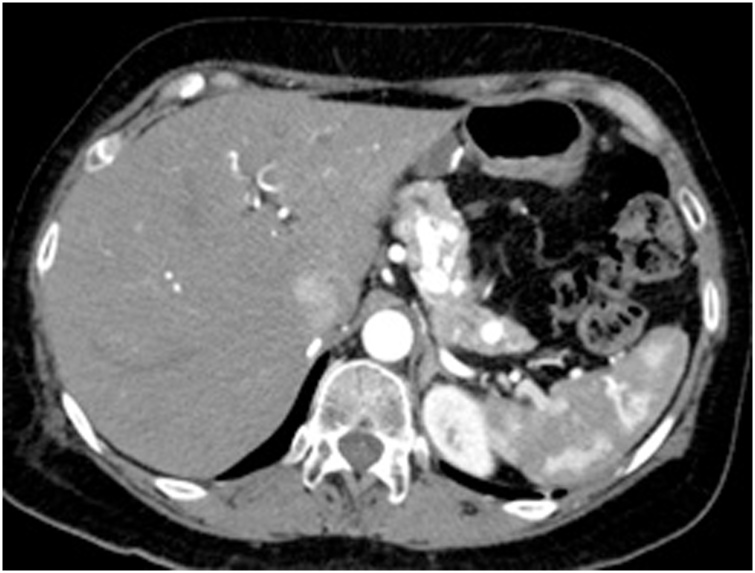
CT scan showing multiple metastatic lesions in the pancreas post Whipple procedure.

We report a case of a 51-year-old female who was diagnosed with clear cell renal cell carcinoma (ccRCC) of the right kidney and underwent radical nephrectomy. The patient was in remission for 6 years post-nephrectomy when she presented with a solitary lesion in the head of the pancreas that was discovered upon surveillance (Fig. 1). It was then resected via a Whipple procedure, interestingly, histopathology reported it as a multi-focal lesion of renal cell carcinoma rather than a solitary lesion (Fig. 2). Ten years after the resection of the primary tumor, the patient presented with a thyroid nodule without any history of thyroid dysfunction. Accordingly, imaging was performed, and a 5.16 cm lesion was found. Subsequently, a fine needle aspiration was done, which revealed RCC metastasis, so the patient underwent a total thyroidectomy (Figs. 3 & 4 ). Six months later, the patient presented with a mass on the anteromedial aspect of the forearm, which was biopsied and confirmed to be yet another metastatic lesion of RCC, and thus it was excised with negative margins (Fig. 5). Within the following year, the patient presented with another mass on the forearm, distal to the site of the previous one. Following the discovery of the subcutaneous lesion on the forearm, metastatic workup was promptly performed, and CT scan showed multiple enhanced pancreatic lesions (Fig. 6). The patient underwent a completion pancreatectomy and resection of the second forearm mass. One year later, another metastatic lesion was found in the left kidney, for which the patient underwent left partial nephrectomy with negative margins. The patient is currently alive and in good condition, as per a recent follow-up.

## 3 Discussion

RCC presents with a classical triad of: palpable mass, flank pain, and hematuria in almost 20% of cases, while in up to 40% of patients it presents with paraneoplastic syndrome. Only a few reported cases presented with metastatic lesions, which were most commonly found in intra-abdominal organs, brain, bones, and lungs [2,4]. RCC can metastasize even after decades of complete excision of the primary tumor, with around 30% of patients presenting with a recurrence years later [5]. It can also metastasize to unusual sites, such as the pancreas. Although the pancreas is an intra-abdominal organ, it is generally perceived as an uncommon site for metastasis, as it was reported that only 2–5% of all metastatic lesions were found in the pancreas and they are usually diffuse throughout the pancreas [6]. Nevertheless, RCC has been reported to be one of the most common malignancies to cause solitary pancreatic metastasis, as reported initially in our case where the patient only had a solitary metastatic lesion in the head of the pancreas. However, this finding might not have been accurate as undetected pancreatic multi-focality has been reported in the literature despite thorough surveillance via PET scan during remission. We suspect that this occurred in our case as her initial PET scan only showed a solitary lesion in the head of the pancreas with atypical enhancement, the histopathology report confirms this theory as it stated the lesion was: *multi-focal metastatic renal cell carcinoma of the clear cell type*. Thus, it is important to have a high index of suspicion for metastatic lesions even in the absence of characteristic radiological findings, and there should not be complete reliance on the regular surveillance protocol [3].

Another rare location for RCC metastasis is the thyroid gland, which was reported to be approximately 1% of the cases. It has also been documented to occur up to 20 years after the resection of the primary tumor. Although it is still not well understood how metastasis targets the thyroid gland, the suggested theory is that it occurs because the thyroid gland is rich with blood supply. Moreover, abnormal thyroid gland has been reported in the literature to be more susceptible to metastasis, which was evident in our patient as the histopathology showed evidence of subclinical thyroiditis [2,5].

The rarest site in our patient’s presentation was the subcutaneous mass found on the forearm. Multiple studies reported that about 3% of renal tumors metastasized to the skin [7]. Skin metastasis of RCC can mimic common dermatological disorders, i.e. lipoma, thus rendering it difficult to be identified due to the low suspicion index. The low suspicion may also be attributed to the fact that the pathogenesis of the skin lesion is usually not directly linked to the primary tumor, as in most cases, there is a long time intervals between the resection of the primary tumor and the metastatic skin lesion. Males were more likely to develop skin metastasis, and the most common areas were the head, neck, and trunk, respectively. Metastasis to the skin is generally regarded as a poor prognostic sign with the survival rate being less than six months. This may be attributed to the fact that 99% of presentations were associated with synchronous visceral metastasis [2].

## 4 Conclusion

RCC is one of the most aggressive malignancies, hence, a high index of suspicion is crucial to detect its metastasis. Even with the right surveillance protocols, some lesions might be missed because they lack the classical radiological findings or are found in atypical locations.

## Conflicts of interest

No conflict of interest applicable for this submission.

## Sources of funding

Funding was not required. No sources exist.

## Ethical approval

This case report is exempt from obtaining an IRB approval.

## Consent

Written informed consent was obtained from the patient for publication of this case report and accompanying images. A copy of the written consent is available for review by the Editor-in-Chief of this journal on request.

## Author contribution

Authors S. Algarni, and M. Mashhour contributed to the paper by collecting all important data and information pertaining to the case.

Authors A. Al Abdrabalnabi, A. AlQattan, and S. Algarni contributed to the paper by reviewing all the available literature related to the case.

Authors M. Al-Qahtani, A. Al Abdrabalnabi, and A. AlQattan contributed by writing and reviewing the final manuscript.

## Registration of research studies

Not applicable.

## Guarantor

Alaa A. Al Abdrabalnabi, Abdullah S. ALQattan, Shahad Algarni, Miral Mashhour, Mohammed AL-Qahtani.

## Provenance and peer review

Not commissioned, externally peer-reviewed.
